# Trends and socio-economic inequalities in overweight- and obesity-related premature cardiovascular disease mortality in Australia

**DOI:** 10.1186/s12916-025-04557-2

**Published:** 2026-01-29

**Authors:** Tim Adair

**Affiliations:** Nossal Institute for Global Health, Melbourne School of Population and Global Health, The University of Melbourne, Level 2, 32 Lincoln Square North, Melbourne, VIC 3010 Australia

**Keywords:** Cardiovascular diseases, Mortality, Obesity, Multiple causes of death, Inequalities, Socio-economic

## Abstract

**Background:**

Australia has previously experienced adverse trends in overweight- and obesity-related cardiovascular disease (CVD) mortality. Its obesity prevalence is relatively high, increasing and shows wide socio-economic inequalities. However, socio-economic inequalities in premature overweight- and obesity-related CVD mortality rate and their trends are unknown. This study measures recent trends in premature overweight- and obesity-related CVD mortality in Australia from 2007 to 2022 and their area-level socio-economic inequalities.

**Methods:**

Premature overweight- and obesity-related CVD mortality was measured as deaths at ages 35–74 years with a CVD reported with at least one (DKOLH-CVD) or two (DKOLH2-CVD) of diabetes, chronic kidney disease, obesity, lipidemias and hypertension. Age-standardised death rates (ASDR) from Australian death registration data were calculated. Inequalities were measured using the Index of Relative Socio-economic Advantage and Disadvantage (IRSAD) and analysed using rate ratios and the Relative Index of Inequality. Obesity prevalence data and their inequalities were also assessed using National Health Survey data.

**Results:**

Premature overweight- and obesity-related CVD mortality, measured as the DKOLH-CVD ASDR, rose from 87.0 (95% confidence interval 84.6–89.5) per 100,000 in 2014 to 103.8 (101.1–106.5) in 2022 for males, or 19%, and from 44.6 (42.9–46.4) in 2013 to 50.5 (48.7–52.4) in 2022 for females, or 13%. When measured as DKOLH2-CVD ASDR, it increased by 37% for males and 21% for females from 2012 to 2022. DKOLH-CVD in ages 35–54 years rose by at least 45% from 2014 to 2022; average obesity prevalence since childhood or young adulthood of these age groups increased by approximately 50% from 2007 to 2022. The ratio of the male DKOLH-CVD ASDR of the most disadvantaged to the most advantaged IRSAD decile increased from 3.16 (2.93–3.41) in 2013–2015 to 3.51 (3.27–3.77) in 2020–2022 and for females from 4.55 (4.08–5.08) to 5.00 (4.51–5.54). Rate ratios were particularly high for DKOLH2-CVD and in ages 35–54 years. Similar socio-economic inequalities were found according to obesity prevalence.

**Conclusions:**

The recent rise in premature overweight- and obesity-related CVD mortality in Australia, especially among those aged 35–54 years and in the most disadvantaged socio-economic deciles, closely mirrors Australia’s increasing obesity prevalence. Failure to effectively tackle Australia’s high obesity prevalence may have a significant detrimental long-term impact on mortality.

**Supplementary Information:**

The online version contains supplementary material available at 10.1186/s12916-025-04557-2.

## Background

There has been a slowdown in the long-term decline in premature mortality rates of cardiovascular disease (CVD) in Australia and several other high-income countries in recent years, a leading cause of premature mortality in these settings [1]. These concerning trends have occurred concurrently with increasing prevalence of overweight and obesity among adult Australian females from 39% in 1990 to 63% in 2021 and males from 52 to 72% [2]. Overweight and obesity increase the risk of both CVD and all-cause mortality, and is the risk factor with the largest contribution to disease burden and the 2nd-largest contribution to premature mortality in Australia [3–5]. Adverse trends in overweight and obesity in Australia have been particularly acute among young- to middle-aged adults, who have experienced relatively high levels of obesity since childhood and for which its negative impacts on health and mortality are especially significant [6–8]. Previous analysis has shown that premature overweight- and obesity-related CVD mortality in Australia, as well as the USA, increased fastest in the mid-2010s among those aged 35–54 years [9].

Australia has a marked socio-economic gradient in the prevalence of overweight and obesity, with it being highest among those with lower completed education and their children and those from regional urban and rural areas [10, 11]. In the USA, there is a large and widening gap in premature CVD mortality rates between people with obesity and who have not completed high school and those who are not obese who have completed a Bachelor’s degree or higher [12]. Given this evidence, coupled with existing large and widening inequalities in premature mortality rates in Australia, measurement of the extent to which premature overweight- and obesity-related CVD mortality rates differ by socio-economic status, and whether they are widening, is of much interest [13]. However, to date there have not been any such published studies in Australia.

This study aims to fill this knowledge gap by measuring trends in premature overweight- and obesity-related CVD mortality in Australia and their inequalities according to area-level socio-economic status, and by assessing whether these inequalities have widened in recent years. It assesses these by sex and age group and compares them to trends and inequalities in obesity prevalence. Since the mid-2010s, when the last such analysis of overweight- and obesity-related premature CVD mortality in Australia was conducted, there has been no sign of a reversal of increasing prevalence in overweight and obesity. Furthermore, the COVID-19 pandemic may have exacerbated existing adverse trends in these mortality rates, such as what occurred in the USA [14].

## Methods

This study used Australian death registration data from 2007 to 2022 in the Australian Bureau of Statistics (ABS) Person Level Integrated Data Asset (PLIDA) [15, 16]. The data comprise all deaths registered in Australia. For each death, the data include all diseases and conditions reported on the death certificate (medical certificate of cause of death) by medical practitioners; this includes the sequence or chain of diseases or conditions leading to death in Part 1, other significant conditions contributing to death in Part 2, and the underlying cause of death that initiated the train of events that led to death. Each disease or condition is coded according to International Classification of Diseases, 10th Revision (ICD-10) [17].

Overweight- and obesity-related premature CVD mortality is defined as deaths occurring at ages 35–74 years where a CVD was reported on the death certificate along with at least one of diabetes (D), chronic kidney disease (K), obesity (O), lipidaemias (L) or hypertension (H), referred to as *DKOLH-CVD*. These conditions are included in the DKOLH-CVD classification irrespective of whether they were reported in Part 1, Part 2 or as the underlying cause of death because previous research has demonstrated that there is subjectivity and inconsistency among physicians as to how deaths involving, for example, diabetes are certified, i.e. whether in the train of events leading to death or as another significant condition [18]. DKOLH-CVD is a definition that has been used in previous studies and represents, i.e. cardiometabolic conditions for which overweight and obesity is a major risk factor [1, 3, 9, 14, 19, 20]. Obesity itself is relatively rarely reported on the death certificate due to differing opinions among physicians as to whether it should be reported as a condition contributing to death or is more a social and behavioural problem [21]. Another metric used was *DKOLH2-CVD*, where at least two of the DKOLH conditions are reported to represent on average a higher level of obesity than DKOLH-CVD. The study also measured *non-DKOLH-CVD*, which is deaths with a CVD but none of the DKOLH causes reported (i.e. CVD mortality not related to overweight and obesity), to compare the different trajectories of sub-components of CVD mortality. More information about ICD-10 codes used is in Additional file 1: Text S1.

The study measured age-standardised death rates (ASDR) at aged 35–74 years calculated using Australian population data and age-standardised to the 2013 Australian population (more detail of population data in Additional file 1: Text S1) [22]. 95% confidence intervals (CI) were calculated assuming a Poisson distribution of deaths. The study calculated the percentage increase of a death rate as:

$$\left(\left(\frac{{DR}_{2}}{{DR}_{1}}\right)-1\right)*100$$

where *DR* is the death rate (either age-specific or age-standardised), *DR*_1_ is the death rate in the first year and *DR*_2_ is the death rate in the second year.

Given that DKOLH-CVD is measured based on any mention of these diseases and conditions on the death certificate, its trends may be biased if there had been an increase in the average number of diseases and conditions being reported on death certificates. This bias was assessed by using a multiple-cause-weighting method to measure cause-specific death rates that controls their trends for any bias due to changes over time in the number of causes reported on a death certificate. The multiple-cause-weighting method apportions fractions of each death to all diseases and conditions reported on the death certificate (*DKOLH-CVD-weighted*) so that they sum to 1.0 for each death [23]. If the trend in DKOLH-CVD was similar to DKOLH-CVD-weighted, then it would not be biased by any change in the likelihood of *all* diseases and conditions being reported on the death certificate. A related source of bias that was assessed was whether there had been any changes over time in the likelihood of decedents who had previously been diagnosed with *DKH-CVD* (a CVD and at least one of diabetes, chronic kidney disease or hypertension; measured in National Health Surveys (NHS) linked to death registration data) who had DKH-CVD reported on their death certificate. If there was no change in this likelihood, the trend in DKOLH-CVD would not be biased. Further detail of these two assessments is shown in Additional file 1: Text S1.

To compare mortality results to obesity trends, the study estimated long-term average obesity prevalence experienced by ages 35–44 years and 45–54 years. Global Burden of Disease (GBD) and NHS data were used to calculate average obesity prevalence from age 15 years onwards for ages 35–44 years and from age 25 years onwards for ages 45–54 years [6]. A starting age of 15 years could not be used for the calculation of obesity prevalence for those aged 45–54 years because people in this age group in 2007 were older than 15 years (18–27 years) in 1980 when data were first available. Further detail of this analyses is described in Additional file 1: Text S1.

Inequalities in death rates were analysed according to the ABS Index of Relative Socio-economic Advantage and Disadvantage (IRSAD), which measures socio-economic conditions of a population within an area, using variables that measure advantage (e.g. percentage of people living in a high-income household) and disadvantage (e.g. percentage of people 15 years and over with no educational attainment) [24]. IRSAD deciles were available in 2013–2022 death registration data for SA2s (Statistical Area Level 2), which have an average population of 10,000 (ranging from 3000 to 25,000 people) [25]. IRSAD deciles were not available in the death registration data for earlier years. For each IRSAD decile, death rates were calculated with population data from the ABS (see Additional file 1: Text S1 for more detail) [26]. Rate ratios to the most advantaged decile (D10) were computed. To quantify relative inequalities in death rates across all IRSAD deciles, the Relative Index of Inequality (RII) was calculated using Poisson regression [27]:

$$\mathrm{ln}\left({D}_{aiy}\right)=\mathrm{ln}\left({P}_{aiy}\right)+{\beta }_{0}+{\beta }_{1}{C}_{aiy}+{\beta }_{2-11}{Age}_{a}$$

where *D* is deaths, *P* is population, *i* is IRSAD decile, *a* is 5-year age group, *y* is year and *C* is the cumulative proportion of the population at each IRSAD decile. The RII is the exponent of the ${\beta }_{1}$ coefficient, and its 95% CI was calculated from the standard error for ${\beta }_{1}$; the RII measures the ratio of the regression-modelled death rate of the most disadvantaged decile to the most advantaged decile. To reduce uncertainty in death rates and RII using IRSAD deciles, each was calculated by combining years as 2013–2015, 2016–2019 and 2020–2022. For analysis of socio-economic inequalities in death rates for specific age groups, we used IRSAD quintiles and calculated death rate ratios compared to the most advantaged quintile (Q5). Again, 95% CIs were calculated assuming a Poisson distribution of deaths.

Age-specific socio-economic inequalities in DKOLH-CVD death rates were compared to obesity prevalence from physical measurement in the NHS 2014–2015 and 2017–2018 combined using IRSAD quintiles. The NHSs measure IRSAD at the SA1 level (Statistical Area Level 1), which has an average population size of about 400 people, ranging from 200 to 800 people [25]. A linear regression for each sex was also conducted to analyse the relationship between age-specific IRSAD quintile ratios for DKOLH-CVD death rates and obesity prevalence.

All data analyses were conducted on the ABS DataLab, where PLIDA data can be accessed. All outputs were approved by the ABS to comply with their rules that each table cell must have a minimum of 10 cases. This meant that categories in some tables could not be disaggregated, such as 35–54 years for some analyses.

## Results

**Fig. 1:**
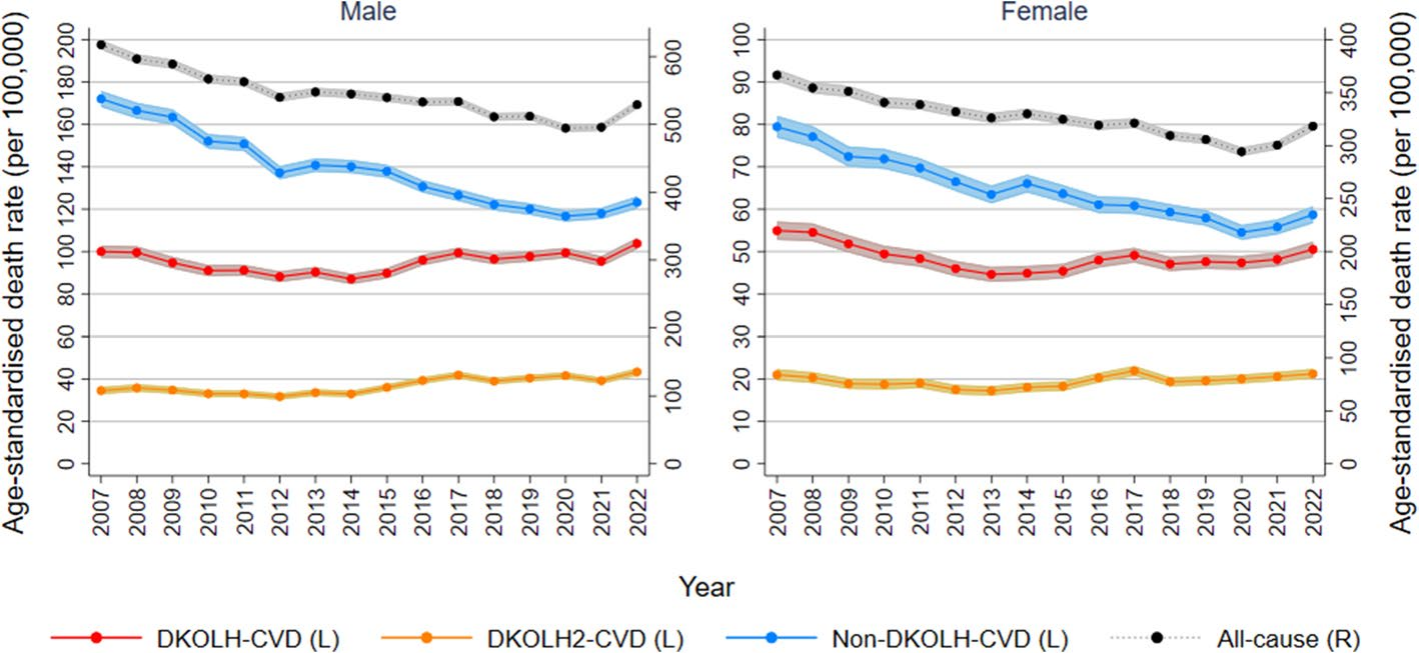
Death rates (age-standardised, per 100,000) by cause, sex and year, 2007–2022. 95% confidence intervals shown in shaded areas. L: left-hand vertical axis. R: right-hand vertical axis

The male DKOLH-CVD ASDR at 35–74 years fell from 100.0 (95% CI 97.0–102.9) per 100,000 in 2007 to 87.0 (84.6–89.5) in 2014, before increasing to 103.8 (101.1–106.5) in 2022, or 19% (Fig. 1). There was a similar trend for females, with a decline from 54.9 (52.8–57.1) in 2007 to 44.6 (42.9–46.4) in 2013, and then a rise to 50.5 (48.7–52.4) in 2022, or 13%. DKOLH2-CVD fell slightly from 2007 to 2012 among both males and females, before rising from 31.7 (30.2–33.3) in 2012 to 43.4 (41.6–45.2) in 2022 for males, or 37%, and 17.5 (16.4–18.6) to 21.2 (20.1–22.4) for females, or 21%. In contrast, from 2007 to 2020 there were steady declines in ASDRs for all causes combined (20% fall in each sex) and especially non-DKOLH-CVD (decline of 32% for males, 31% for females), before an increase in 2021–2022 during the COVID pandemic. For all 2007–2022, deaths with a CVD (i.e. DKOLH-CVD and non-DKOLH-CVD combined) were over 43% of male and 35% of female all-cause deaths at ages 35–74 years.

Of the specific causes that comprise DKOLH-CVD, diabetes, hypertension and chronic kidney disease ASDRs fell from 2007 to the early 2010s before rising thereafter, with rates in 2022 compared with 2007 being higher or at least as high for males and lower for females (Additional file 1: Fig. S1). In contrast, ASDRs for obesity more than doubled and for lipidaemias also rose for both males and females over the period. For both males and females, the trend in DKOLH-CVD ASDRs when weighted for the number of other causes reported on the death certificate was almost identical to the unweighted trend (i.e. the trend presented in the main analyses) (Additional file 1: Fig. S2). The percentage of decedents who had been diagnosed with DKH-CVD before their death and it was reported on their death certificate was almost unchanged from 31.8% (22.7–40.9%) in 2015–2018 to 32.0% (24.3–39.6%) in 2019–2022, but with wide CIs.

**Fig. 2:**
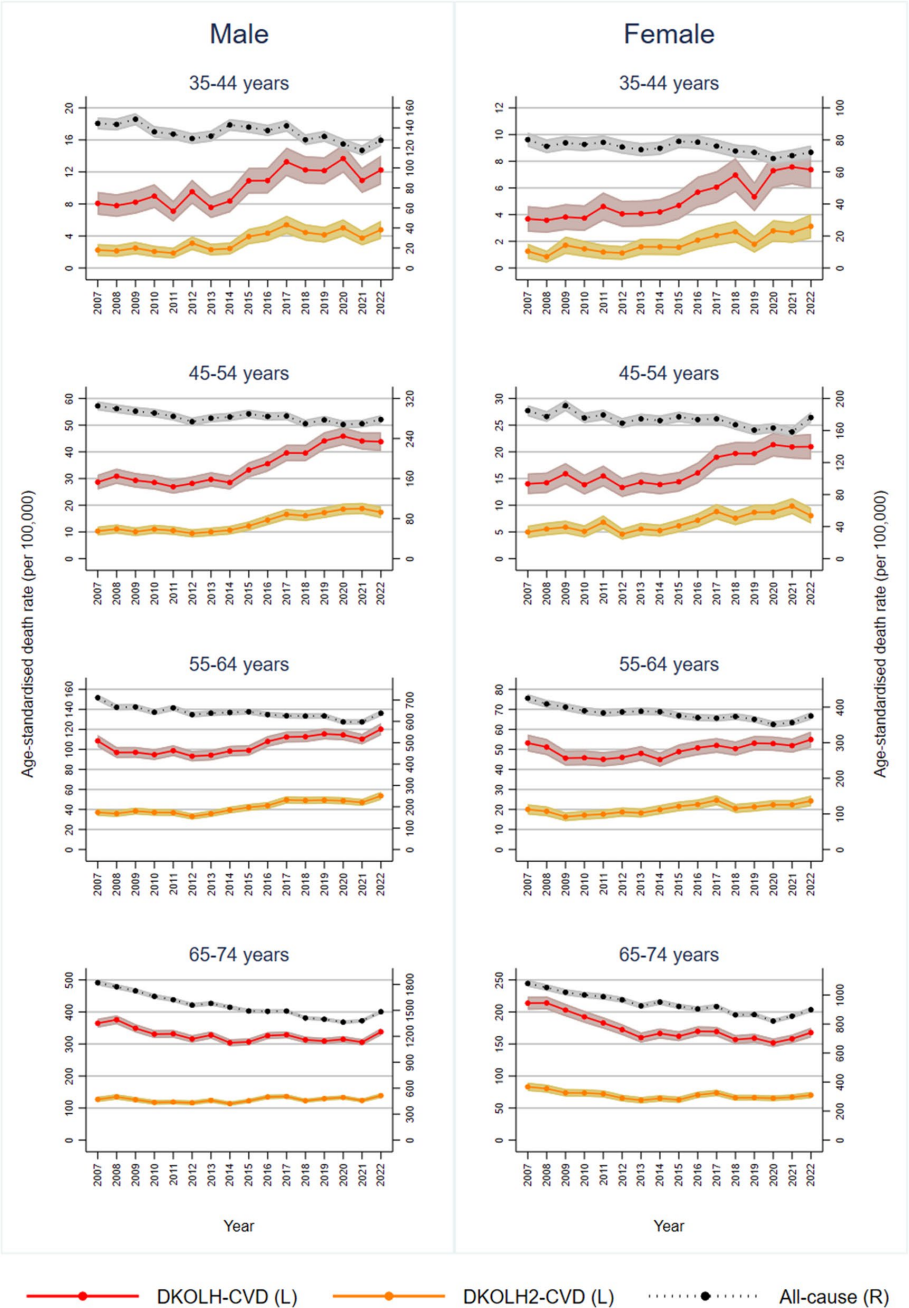
Death rates (age-specific, per 100,000), by cause, sex and year, 2007–2022. 95% confidence intervals shown in shaded areas

The increase in DKOLH-CVD ASDRs was most pronounced at ages 35–44 and 45–54 years (Fig. 2 and Table 1). In each age group, the DKOLH-CVD ASDR was steady from 2007 to 2014 before increasing sharply to 2022, with at least a 4.7% annualised or 44% overall increase in each. For DKOLH2-CVD, there was also an increase in ASDRs that was smaller in absolute but larger in relative terms than for DKOLH-CVD, with at least a 7.1% annualised or 73% overall rise in each age group. These trends contrast with declines in all-cause death rates in each age group, with a small rise in 2022. At ages 55–64 years, there also was an increase in DKOLH-CVD ASDRs from the mid-2010s but not as rapidly as in the younger age groups, while at 65–74 years they fell from 2007 to 2014 and were steady thereafter. In these older age groups, all-cause ASDRs steadily declined.

**Table 1:** Annualised rate of change (%) in death rates (age-specific), by cause and age group, males and females, 2007–2009 to 2012–2014, 2012–2014 to 2020–2022

Age group	Period	Male			Female		
		All-cause	DKOLH-CVD	DKOLH2-CVD	All-cause	DKOLH-CVD	DKOLH2-CVD
35–44	2007–2009 to 2012–2014	− 1.5	+ 1.1	+ 2.6	− 0.9	+ 2.2	+ 2.4
	2012–2014 to 2020–2022	− 1.1	+ 4.7	+ 7.1	− 0.8	+ 7.6	+ 9.0
45–54	2007–2009 to 2012–2014	− 1.4	− 0.6	− 0.9	− 1.4	− 1.2	− 1.3
	2012–2014 to 2020–2022	− 0.3	+ 5.6	+ 7.7	− 0.5	+ 5.4	+ 7.1
55–64	2007–2009 to 2012–2014	− 1.3	− 1.1	− 0.6	− 1.2	− 1.5	+ 0.5
	2012–2014 to 2020–2022	− 0.5	+ 2.4	+ 4.1	− 0.9	+ 1.8	+ 2.4
65–74	2007–2009 to 2012–2014	− 2.5	− 2.8	− 1.8	− 2.0	− 4.6	− 4.1
	2012–2014 to 2020–2022	− 1.3	+ 0.1	+ 1.4	− 1.2	− 0.5	+ 0.6

**Fig. 3:**
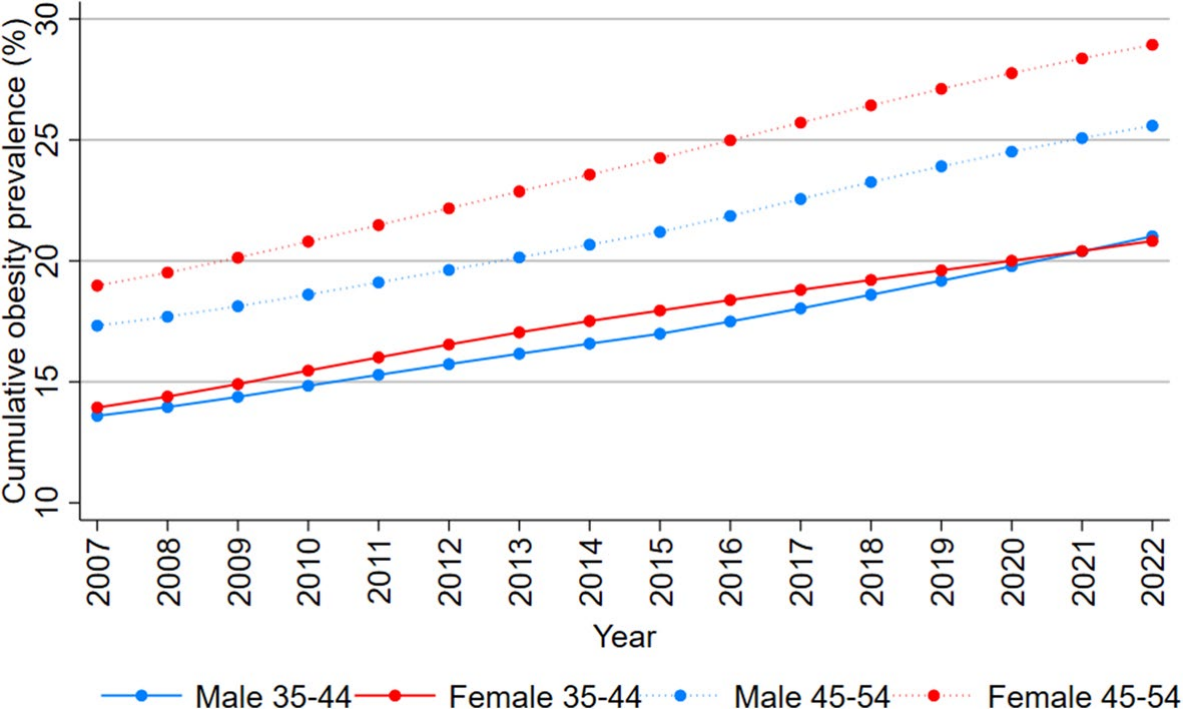
Average obesity prevalence (%) (since age 15 years for people aged 35–44 years and since age 25 years for people aged 45–54 years), by sex and year, 2007–2022. Note: Average obesity prevalence for age 35–44 years is based on the obesity prevalence experienced by each single year birth cohort within that age group for that year (born 1963 to 1972 for 2007) from age 15 years onwards. For 45–54 years, it is based on age 25 years onwards. A starting age of 15 years could not be used for the calculation of obesity prevalence for those aged 45–54 years because data only available from 1980. Data sources: GBD Obesity Collaborators, NHS 2011–2012, 2014–2015, 2017–2018, 2022 [6]

Figure 3 shows the average obesity prevalence from ages 15 years onwards for those aged 35–44 years, and from 25 years onwards for those aged 45–54 years, rose consistently from 2007 to 2022 by about 50% for each category.

**Fig. 4:**
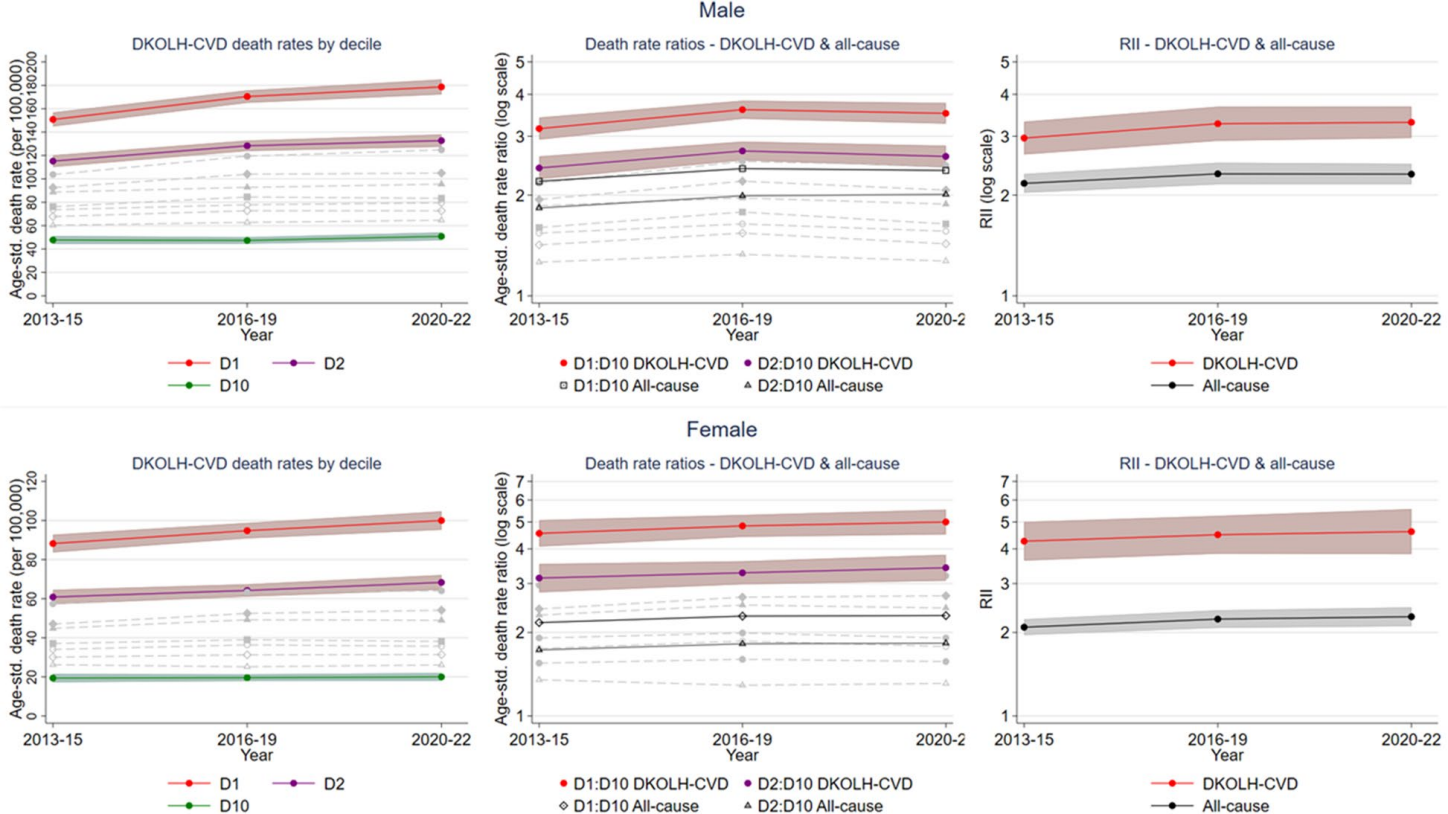
DKOLH-CVD death rate (age-standardised, per 100,000) by IRSAD decile and DKOLH-CVD and all-cause death rate ratios and Relative Index of Inequality (RII), by sex and year (grouping), 35–74 years, 2013–2022. RII is calculated using IRSAD decile. 95% confidence intervals shown in shaded area. Dashed lines in grey are DKOLH-CVD death rates for other deciles or decile ratios (relative to D10)

In the most disadvantaged IRSAD decile (D1), the DKOLH-CVD ASDR for males rose by 18% from 2013–2015 to 2020–2022, with the rate ratio to the most advantaged decile (D10) increasing from 3.16 (2.93–3.41) in 2013–2015 to 3.60 (3.38–3.83) in 2016–2019 and then falling slightly to 3.51 (3.27–3.77) in 2020–2022 (Fig. 4). The RII also rose but with overlapping CIs. For females, the DKOLH-CVD ASDR in D1 increased by 13%, and the ratio to D10 was higher than for males, increasing from 4.55 (4.08–5.08) to 5.00 (4.51–5.54) from 2013–2015 to 2020–2022. The RII also increased, but again with relatively wide CIs. All-cause rate ratios and RIIs were considerably lower than for DKOLH-CVD.

**Fig. 5:**
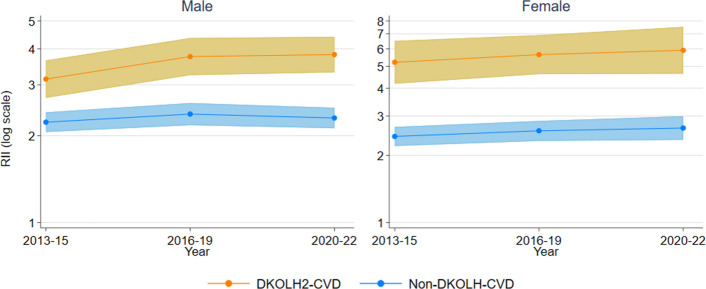
DKOLH2-CVD and non-DKOLH-CVD death rate (age-standardised, per 100,000) Relative Index of Inequality (RII), by sex and year (grouping), 35–74 years, 2013–2022. RII is calculated using IRSAD decile. 95% confidence intervals shown in shaded area

There was also a sharp increase in DKOLH2-CVD ASDRs in D1 for males of 28% and females of 17% from 2013-15 to 2020-22 (Additional file 1: Fig. S3). The DKOLH2-CVD RII for males and females was higher than DKOLH-CVD later in the period (Fig. 5). The non-DKOLH-CVD RII for males was much closer to that for all causes than DKOLH-CVD).

**Fig. 6:**
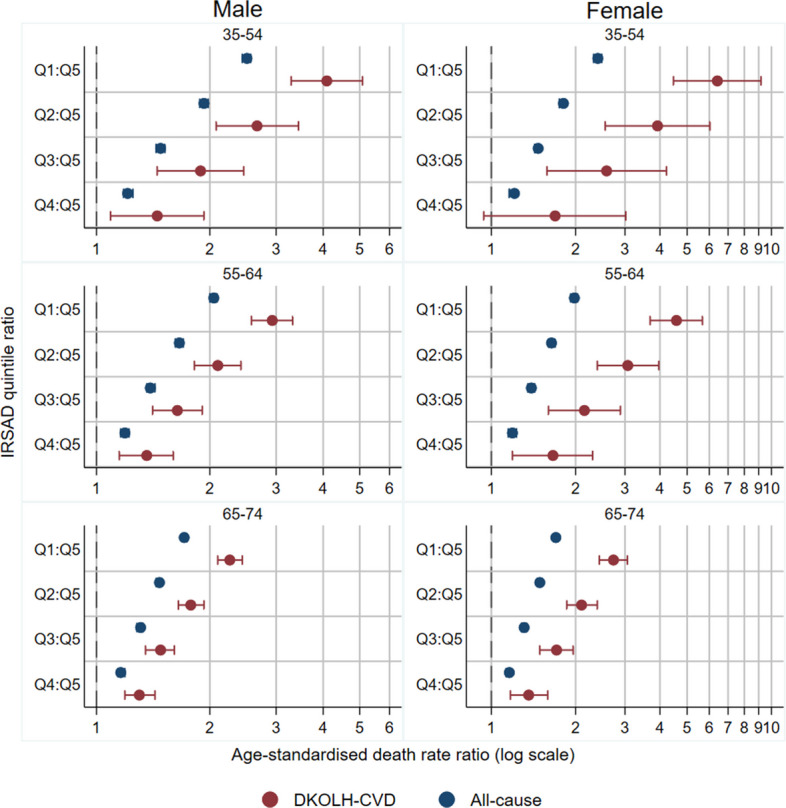
DKOLH-CVD and all-cause death rate ratio (age-standardised) by sex, age group and IRSAD quintile, 2013–2022 combined. 95% confidence intervals shown in bars. The 35–54 years age group could not be disaggregated further because of ABS output rules

Death rate ratios by IRSAD quintile for DKOLH-CVD were widest at ages 35–54 years, reaching 6.41 (4.47–9.18) for the most disadvantaged (Q1) to most advantaged quintile (Q5) for females and 4.09 (3.29–5.09) for males (Fig. 6). All-cause rate ratios were much lower than for DKOLH-CVD, especially at 35–54 years, where they were about 2.5.

**Fig. 7:**
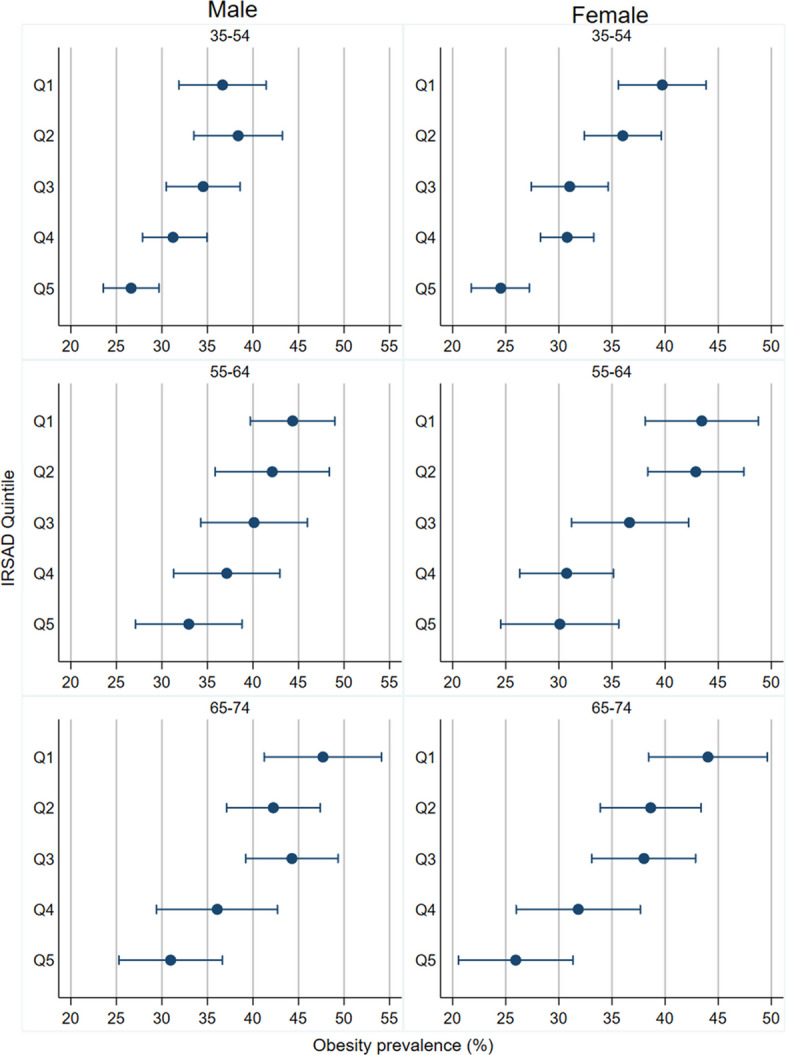
Obesity prevalence (%) by sex, age group and IRSAD quintile, NHS 2014–2015 and 2017–2018 combined. Bars show 95% confidence intervals

Differences in obesity prevalence by IRSAD quintile for each age group were also found (Fig. 7), with Q1 and Q2 consistently having much higher obesity prevalence than Q5. These ratios for obesity prevalence are lower than for mortality when presented on the same chart, likely because obesity prevalence is much more common than death (Additional file 1: Fig. S4). However, sex-specific linear regressions of these ratios of the DKOLH-CVD death rate and obesity prevalence rate show a positive relationship (Additional file 1: Table S1).

## Discussion

Premature overweight- and obesity-related CVD mortality rates, represented by DKOLH-CVD, increased in Australia from the mid-2010s to 2022 to be higher than in 2007, with an even faster relative rise where two or more DKOLH conditions were reported (DKOLH2-CVD). The declines in premature CVD mortality without any DKOLH conditions reported (non-DKOLH-CVD) demonstrate a changing composition of CVD mortality in Australia towards cardiometabolic conditions for which overweight and obesity are a major risk factor. Existing data from Australia suggest a significant increase in chronic kidney disease prevalence from 2010 to 2020, especially in stages 3 and above, a sharp rise in diabetes prevalence to the mid-2010s followed by a slight decline, which likely correlates to the trend in mortality given a time lag, and no change in uncontrolled blood pressure during the 2010s [28–30]. For both sexes, there was a sharp increase in reporting of obesity on the death certificate which, although it is relatively rarely reported, demonstrates its increasing importance in contributing to death. The rise in premature overweight- and obesity-related CVD mortality in 2007–2022 was fastest at younger ages, which correspond with increases in obesity prevalence in these age groups since childhood/young adulthood. These trends are not unexpected given that experiencing obesity from childhood or young adulthood is associated with higher mortality in later life through higher prevalence of cardiometabolic conditions [31]. The DKOLH-CVD and DKOLH2-CVD mortality rates are increasing particularly quickly in the most disadvantaged deciles with large and widening inequalities. The largest disparities occur at younger age groups and correspond with high socio-economic inequalities in obesity prevalence. This finding is consistent with previous research showing disparities in prevalence of chronic kidney disease, diabetes and to a lesser extent hypertension [28–30]. The results also contrast with much lower inequalities for all-cause and non-DKOLH-CVD mortality, demonstrating the distinct disparities of overweight- and obesity-related CVD mortality.

The trends and disparities in overweight- and obesity-related CVD mortality, especially at ages 35–54 years, could have a significant detrimental impact on all-cause mortality and life expectancy in Australia should these continue to older ages where CVD accounts for a higher proportion of deaths. These results are very concerning given the role of obesity in contributing to slowing life expectancy improvements in the USA, as well as Australia’s sluggish growth in life expectancy in the past decade, relatively poor mortality trends at ages less than 50 years, high obesity prevalence and existing widening inequalities in premature mortality [13, 32–34]. Obesity is a major public health challenge globally, especially given that it has continued to increase for several decades with limited effectiveness of existing interventions, aside from bariatric surgery and potentially sugar-sweetened beverage taxes and trans-fat policies [2, 35]. Numerous factors lead to rising obesity, as recognised by the Australian *National Obesity Strategy 2022–2032*, including an increase in intake of calories, ultra-processed and unhealthy food and drink with high sugar and saturated/trans fats; food and drink that is widely advertised, affordable and convenient for a time-poor society while healthy options can be inaccessible in some lower socio-economic areas [2, 36–38]. Australian policy on diet has been criticised as falling well short of international best practice, being based on short-term thinking and influenced by vested interests [39]. There has also been a lack of improvement in physical activity in Australia, with the physical environment and urban design identified as reliant on motor vehicle transport and lacking walkability, which help lead to large socio-economic inequalities exist in physical activity [36, 37, 39]. The role of obesogenic environments in affecting obesity is particularly relevant for younger adults, many of whom have been exposed to these for their entire lives [37]. For low socio-economic populations, there are also stressors they disproportionately experience that affect cardiometabolic conditions, including loneliness, marital stress and workplace stress, either physiologically or through unhealthy coping behaviours [37].

Recommendations to reduce obesity should include evidence-based treatments for people with obesity (e.g. bariatric surgery) and interventions to improve accessibility to healthy food, reduce fast food advertising and facilitate more physical activity by addressing the obesogenic environment and improving access to care; these should be underpinned by collaborations between multiple sectors, led by the health sector [36, 37]. However, this is challenging given the complexity of factors influencing obesity, and that losing and maintaining weight loss is difficult in the absence of medication, as reflected by Australia’s *National Obesity Strategy 2022–2032* only aiming to prevent further rises in obesity by 2030 [36].

In recent years, the demonstrated effectiveness and rise in usage of semaglutide drugs has led to speculation of its importance in addressing obesity. These were originally developed as diabetes medication but have led to reductions in weight, CVD and other chronic diseases, with the US Food and Drug Administration’s approving the semaglutide Wegovy for heart disease treatment [35, 40]. However, concerns about semaglutide drugs as a long-term effective means to reduce obesity relate to its cost, accessibility, adherence over the long-term and potential side effects [35, 41, 42]. In Australia, no anti-obesity medications are subsidised by the Pharmaceutical Benefits Scheme (PBS); Ozempic is subsidised only for management of type II diabetes and not weight loss while Wegovy is approved by the Therapeutic Goods Administration for those with CVD and high body mass index but is not PBS-subsidised [41, 42]. There are plausible concerns that focusing on these drugs may exacerbate the existing inequalities in obesity and hence mortality, and hence should not remove focus from broader public health efforts given they will not impact the structural causes of high and increasing obesity [2, 35].

A limitation of the study is that it uses an indirect measure of overweight- and obesity-related CVD mortality of DKOLH-CVD that represents a group of conditions for which it is evidenced as being a risk factor, while not having direct evidence of the body mass index of the deceased individuals aside from the relatively rare reporting of obesity on the death certificate [3, 19, 20]. However, the strong correlation of obesity prevalence and DKOLH-CVD (and DKOLH2-CVD) mortality trends and differentials by age and area socio-economic status provide further support for this metric being a valuable means of measuring overweight- and obesity-related CVD mortality. Further, the use of multiple cause of death data in this study enables granular analyses of trends and differentials by age and socio-economic status such as shown in the results here. Additional analysis could identify those diseases and conditions with the highest likelihood of being reported on the death certificate for those who are obese by using the NHS linked to death registration. Potential bias in the DKOLH-CVD measure could be due to changing practices of it being reported on the death certificate; however, there was no clear evidence of this in the analyses, whether increases in the average number of reported conditions or a rising likelihood of it being a reported cause of death where the decedent had been diagnosed with any of these conditions, although the low sample for the latter prevents narrower CIs. Finally, the measurement of socio-economic inequalities was undertaken at the area-level, which may under-estimate inequalities at the individual-level; in future these could be measured with linked census and death registration data [15].

## Conclusions

This study has shown that premature overweight- and obesity-related CVD mortality in Australia has been increasing in recent years, especially among those aged 35–54 years, with particularly sharp increases in the most disadvantaged socio-economic deciles and large and widening disparities compared with the most advantaged deciles. These trends and differentials closely mirror the Australia’s increasing obesity prevalence and its wide inequalities. As stated by the *National Obesity Strategy*, failure to address high obesity means Australia faces higher levels of weight-related chronic diseases and premature death in future [36].

## Supplementary Information

Additional file 1: Text S1 Further detail on methods. Fig. S1 Individual components of DKOLH-CVD death rates (age-standardised, per 100,000) by sex, 2007–2022. Fig. S2 DKOLH-CVD and DKOLH-CVD-weighted comparison of death rate trends, by sex, 2007–2022. Fig. S3 DKOLH2-CVD death rate (age-standardised, per 100,000) by sex and IRSAD decile, 2013–2022. Fig. S4 Ratio of all-cause mortality death rate and DKOLH-CVD death rate (2013–2022 combined) and obesity prevalence rate to IRSAD quintile Q5 (NHS 2014–2015 and 2017–2018 combined), by sex and age. Table S1 Results of linear regressions of log of ratio of the DKOLH-CVD death rate (to IRSAD quintile 5) and log of ratio of the obesity prevalence rate (to IRSAD quintile 5).

## Data Availability

The data underlying this article cannot be shared publicly because they can only be accessed by approved users of PLIDA via the ABS DataLab.

## Abbreviations

ABS: Australian Bureau of Statistics
ASDR: Age-standardised death rates
CI: Confidence intervals
CVD: Cardiovascular disease
DKH-CVD: A CVD and at least one of diabetes, chronic kidney disease or hypertension reported
DKOLH-CVD: Where a CVD was reported on the death certificate along with at least one of diabetes (D), chronic kidney disease (K), obesity (O), lipidaemias (L) or hypertension (H)
DKOLH-CVD-weighted: Measure of DKOLH-CVD that uses a multiple-cause-weighting method that apportions fractions of each death to all diseases and conditions reported on the death certificate
DKOLH2-CVD: Where at least two of the DKOLH conditions are reported
D1: Most disadvantaged IRSAD decile
D5: Most advantaged IRSAD decile
GBD: Global Burden of Disease
ICD-10: International Classification of Diseases, 10th Revision
IRSAD: Index of Relative Socio-economic Advantage and Disadvantage
NHS: National Health Survey
Non-DKOLH-CVD: Deaths with a CVD but none of the DKOLH causes reported
PBS: Pharmaceutical Benefits Scheme
PLIDA: Person Level Integrated Data Asset
Q1: Most disadvantaged IRSAD quintile
Q5: Most advantaged IRSAD quintile
RII: Relative Index of Inequality
SA1: Statistical Area Level 1
SA2: Statistical Area Level 2
