# Supplementary material for: Trends and socio-economic inequalities in overweight- and obesity-related premature cardiovascular disease mortality in Australia
Source: BMC Med. 2026 Jan 29;24:3. doi: 10.1186/s12916-025-04557-2 (PMC12857031; doi:10.1186/s12916-025-04557-2)
Supplement: Supplementary file 1 — Additional file 1: Text S1 Further detail on methods. Fig. S1 Individual components of DKOLH-CVD death rates (age-standardised, per 100,000) by sex, 2007–2022. Fig. S2 DKOLH-CVD and DKOLH-CVD-weighted comparison of death rate trends, by sex, 2007–2022. Fig. S3 DKOLH2-CVD death rate (age-standardised, per 100,000) by sex and IRSAD decile, 2013–2022. Fig. S4 Ratio of all-cause mortality death rate and DKOLH-CVD death rate (2013–2022 combined) and obesity prevalence rate to IRSAD quintile Q5 (NHS 2014–2015 and 2017–2018 combined), by sex and age. Table S1 Results of linear regressions of log of ratio of the DKOLH-CVD death rate (to IRSAD quintile 5) and log of ratio of the obesity prevalence rate (to IRSAD quintile 5). [file 12916_2025_4557_MOESM1_ESM.docx]

**Trends and socio-economic inequalities in overweight- and obesity-related premature cardiovascular disease mortality in Australia**

**Additional File 1**

**Text S1: Further detail on methods**

*ICD-10 codes used*

DKOLH-CVD is measured as deaths where a cardiovascular disease (ICD-10 codes I00-I99) is reported on the death certificate (excluding cardiac arrest (I46), a more immediate cause of death which can be unrelated to cardiovascular diseases) and one or more of the following conditions for which overweight and obesity increase the risk of dying from: diabetes (E10-E14), chronic kidney diseases (N18), obesity (E65-E66), lipidemias (E78) or hypertension (I10-I13).

*Assessment of potential biases in DKOLH-CVD trends*

Trends in DKOLH-CVD may be biased if there has been an increase in the number of diseases and conditions being reported on the death certificate. The study used a multiple-cause-weighting method that apportions fractions of each disease and condition based on all deaths reported on the death certificate; the underlying cause of death is given a score of 0.5 and each remaining cause is given a score of 0.5 divided by the number of remaining reported diseases and conditions.(23) The sum of scores for each death equals 1.0 This is referred to as *DKOLH-CVD-weighted*. If the trend in DKOLH-CVD-weighted is similar to DKOLH-CVD, then any change in the likelihood of *all* diseases and conditions being reported on the death certificate over the period did not bias DKOLH-CVD trends.

However, DKOLH-CVD-weighted will not measure whether the study measures whether there have been any changes in the likelihood of a decedent who had been diagnosed with overweight- and obesity-related CVD having it reported on their death certificate. To measure this, the study took advantage of linkage of death registration with reported disease diagnosis data in the Australian National Health Survey (NHS), a nationally representative sample survey of the health of the Australian population, conducted by the ABS. The analysis was restricted to people in the 2014-15 and 2017-18 NHSs who reported that they had previously been diagnosed with a CVD and either diabetes, chronic kidney disease or hypertension (DKH-CVD), and who subsequently died.(2, 3) From this population, the percentage that had DKH-CVD reported on their death certificate was calculated. Obesity and lipidemias were excluded because they are the rarest of the DKOLH-CVD causes and may not be recognised as diseases of conditions that had been diagnosed; these would have only added 10 more cases if they were included. The percentage was compared for deaths in 2015-18 and 2019-22; deaths for earlier years could not be analysed because the earliest NHS able to be linked to death registration was 2014-15. The NHS and death registration data were linked in in the Australian Bureau of Statistics (ABS) Person Level Integrated Data Asset (PLIDA) via a Personal Linkage Spine.(15) Linkage rates with the Personal Linkage Spine were 98.5% of deaths from July 2015-December 2022 in the death registration dataset, 94.9% of 2014-15 NHS cases and 91.7% of 2017-18 NHS cases.(15) 95% confidence intervals were calculated using the replicate weights technique, as recommended by the ABS (3). Given small number of cases (only 321 decedents had a DKH-CVD diagnosis), all ages combined were analysed.

*Obesity prevalence data and methods*

Calculation of average cohort obesity prevalence requires single-year-of-age age-specific obesity prevalence data for each calendar year. The Global Burden of Disease (GBD) published obesity prevalence by five-year age group from 1980-2015.(6) We interpolated obesity prevalence by single year of age from these data. We complemented these data with obesity prevalence measured from the 2011-12, 2014-15, 2017-18 and 2022 NHSs (6-9). Obesity was measured as based on body mass index (BMI) measurements collected of NHS participants; a BMI of greater than or equal to 30kg/m^2^ was defined as obese. The 2011-12 NHS was conducted of 20,500 respondents, the 2014-15 NHS was conducted of 19,259 respondents, the 2017-18 NHS of 21,315 respondents and the 2022 NHS of 17,073 respondents (2, 3, 6, 7). We again interpolated prevalence by single year of age, this time from estimates for 18-24 years and then 10-year age groups. To ensure consistency of NHS and GBD obesity prevalence, we calculated the age-sex-specific ratio of NHS 2011-12 and 2014-15 obesity prevalence to GBD obesity prevalence and applied these ratios to adjust age-sex-specific rates from the NHS for years from 2016-22. We interpolated 2015-22 obesity prevalence for years between NHSs. For 1978-79, when the 1963 and 1964 birth cohorts (aged 43-44 in 2007) were aged 15-16, and the 1953 and 1954 birth cohorts (aged 53-54 in 2007) were aged 25-26, we assumed the same age-sex-specific obesity prevalence as in 1980.

Average obesity prevalence was calculated from age 15 years onwards for age 35-44 years and from age 25 years onwards for age 45-54 years. For example, people aged 35-44 years in 2007 were born from 1963 (age 44) to 1972 (age 35). For each single year cohort, obesity prevalence that the cohort experienced for each single year of age from 15 years onwards was calculated. The 1972 birth cohort, for example, would have been aged 15 in 1987 and so we used the obesity prevalence of 15 years in 1987, 16 years in 1988, and so on. The average obesity prevalence of people aged 35-44 years in 2007 was calculated from the average obesity prevalence of each single year birth cohort 1963 to 1972 for each age from 15 years until their age in 2007 (44 to 35). A starting age of 15 years could not be used for the calculation of obesity prevalence for those aged 45-54 years because people in this age group in 2007 were older than 15 years (18-27 years) in 1980 when data were first available.

*Population data*

Population data are form the Australian Bureau of Statistics.(22) It is the estimated resident population, which includes all people who usually live in Australia, irrespective of citizenship and visa status. (22) The population estimates are calculated from 2021 Census data and updated every quarter with estimated births, deaths, overseas and interstate migration. The population data for Index of Relative Socio-economic Advantage and Disadvantage (IRSAD) deciles was calculated from the same estimated resident population estimates for Statistical Area Level 2 (SA2), which were then matched to the IRSAD decile of that SA2 and then aggregated.(26) The population at 30 June was used as the denominator for each year.

**Figure S1: Individual components of DKOLH-CVD death rates (age-standardised, per 100,000) by sex, 2007-22**

95% confidence intervals shown in shaded area.

**Figure S2: DKOLH-CVD and DKOLH-CVD-weighted comparison of death rate trends, by sex, 2007-22**

95% confidence intervals shown in shaded area. *DKOLH-CVD weighted* is the weighted for the number of other causes reported in the death certificate. *DKOLH-CVD unweighted* is used in all other analyses in this study.

**Figure S3: DKOLH2-CVD death rate (age-standardised, per 100,000) by sex and IRSAD decile, 2013-22**

95% confidence intervals shown in shaded area. Dashed lines in grey are DKOLH2-CVD death rates for other deciles.

**Figure S4: Ratio of all-cause mortality death rate and DKOLH-CVD death rate (2013-22 combined) and obesity prevalence rate to IRSAD quintile Q5 (NHS 2014-15 and 2017-18 combined), by sex and age**

Derived from Figures 6 and 7.

**Table S1: Results of linear regressions of log of ratio of the DKOLH-CVD death rate (to IRSAD quintile 5) and log of ratio of the obesity prevalence rate (to IRSAD quintile 5)**

| **Sex** | **Coefficient (β_1_) of log of obesity prevalence ratio (95% CI)** | **Model r-squared** |
| --- | --- | --- |
| Male | 3.09 (1.63-4.55) | 0.79 |
| Female | 2.80 (1.47-4.13) | 0.81 |

n=12. CI: Confidence interval

Each regression was conducted for each sex, with each case being a quintile-specific ratio. Outcome variable: natural logarithm of the ratio of the DKOLH-CVD death rate for an IRSAD quintile to quintile 5. Covariates: natural logarithm of the ratio of obesity prevalence for an IRSAD quintile to quintile 5 and age group.
